# NanoMiner — Integrative Human Transcriptomics Data Resource for Nanoparticle Research

**DOI:** 10.1371/journal.pone.0068414

**Published:** 2013-07-12

**Authors:** Lingjia Kong, Soile Tuomela, Lauri Hahne, Helena Ahlfors, Olli Yli-Harja, Bengt Fadeel, Riitta Lahesmaa, Reija Autio

**Affiliations:** 1 Department of Signal Processing, Tampere University of Technology, Tampere, Finland; 2 Turku Centre for Biotechnology, University of Turku and Åbo Akademi University, Turku, Finland; 3 Turku Doctoral Programme of Biomedical Sciences, Turku, Finland; 4 Division of Molecular Toxicology, Institute of Environmental Medicine, Karolinska Institutet, Stockholm, Sweden; National Institute of Health (NIH), United States of America

## Abstract

The potential impact of nanoparticles on the environment and on human health has attracted considerable interest worldwide. The amount of transcriptomics data, in which tissues and cell lines are exposed to nanoparticles, increases year by year. In addition to the importance of the original findings, this data can have value in broader context when combined with other previously acquired and published results. In order to facilitate the efficient usage of the data, we have developed the NanoMiner web resource (http://nanominer.cs.tut.fi/), which contains 404 human transcriptome samples exposed to various types of nanoparticles. All the samples in NanoMiner have been annotated, preprocessed and normalized using standard methods that ensure the quality of the data analyses and enable the users to utilize the database systematically across the different experimental setups and platforms. With NanoMiner it is possible to 1) search and plot the expression profiles of one or several genes of interest, 2) cluster the samples within the datasets, 3) find differentially expressed genes in various nanoparticle studies, 4) detect the nanoparticles causing differential expression of selected genes, 5) analyze enriched Kyoto Encyclopedia of Genes and Genomes (KEGG) pathways and Gene Ontology (GO) terms for the detected genes and 6) search the expression values and differential expressions of the genes belonging to a specific KEGG pathway or Gene Ontology. In sum, NanoMiner database is a valuable collection of microarray data which can be also used as a data repository for future analyses.

## Introduction

Engineered nanoparticles (ENs) have been specifically manufactured to be incorporated into a product or process, *i.e.*, engineered to meet specific needs. Their applications cover both engineering and biomedical sectors (*e.g.* in drug delivery and gene therapy). There are more than 100,000 ENs with differences in their shape, size, surface and chemical composition [1]. Development and manufacturing of ENs are expanding at an accelerating pace because of the novel characteristics of ENs and their promising applications. On the other hand, the increasing use of ENs has raised the need to assess their potential benefits and risks [2].

Numerous recent studies have reported a variety of biological and toxicological interactions of ENs in *in vitro* and *in vivo* experimental systems [1], [3]. Microarray technology is a powerful tool and may enhance our understanding of underlying mechanisms of toxicity, thus providing extensive information upon which to base public health and regulatory decisions [4]–[6]. Since microarray technology is becoming more efficient and affordable, increasing numbers of EN-related transcriptomic experiments are being performed each year. As a result, experimental data from EN-related microarray studies is accumulating in public databases. For the benefit of researchers, it would be useful for this information to be gathered, curated, and stored in a central repository as well as a set of recommended experimental criteria created and disseminated.

As an initial step to reach this goal, we have developed NanoMiner, a database containing experimental results from different nanoparticle related gene expression microarray studies. In the public databases such as Gene Expression Omnibus (GEO) [7] or ArrayExpress [8] there are hundreds of datasets of transcriptomics data from all fields of science. In NanoMiner, the nanoparticle related data derived from *in vitro* studies is extracted from these databases and processed consistently across each dataset facilitating data access, exploration, and retrieval, as well as comparison between different studies. In addition, NanoMiner provides links to the original studies and an access to the annotations of the data samples. NanoMiner also has various visualization and statistical analysis options to aid nanoparticle research. With the wide selection of its data analysis and illustration options, NanoMiner is a unique tool for researchers working in toxicogenomics, which can be used, for example, to anticipate the outcome of the interaction of nanoparticles with biological systems and thus the future risk of using these materials.

## Results

**Figure 1:**
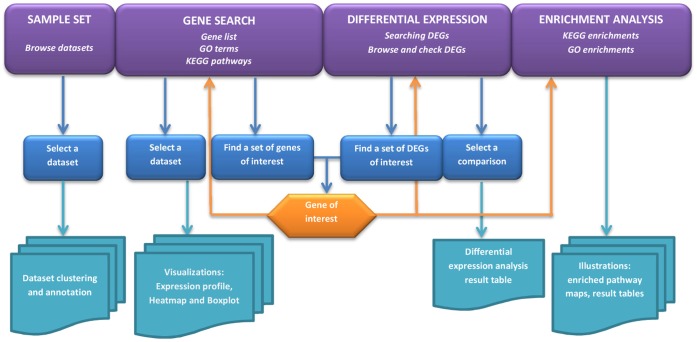
NanoMiner workflow diagram. NanoMiner provides several options to use the database: [1] SAMPLE SET: In the Sample Set section of the database it is possible to browse the datasets and cluster the samples within the datasets. In addition, in this section it is possible to download all the annotations of the dataset. [2] GENE SEARCH: In the Gene Search page the user can search a gene or set of genes based on the gene names on the Gene Ontology (GO) or KEGG pathways. Further, the user can plot gene expression profiles within a given dataset. There are three options for plotting the genes; expression values of all selected genes in one plot, heatmap of all selected gene values, or boxplot of each gene separately. [3] DIFFERENTIAL EXPRESSION: In the Differential Expression page, the user can find the differentially expressed genes (DEGs) in the pre-computed comparisons. In addition, it is possible to search for differentially expressed genes across several datasets by first selecting genes of interest and then analyzing their expression over the comparisons. [4] ENRICHMENT ANALYSIS: the user can identify the enriched KEGG-pathways and GO classes for the detected genes. Further, the enriched KEGG pathways can be illustrated by pathway maps.

The NanoMiner database includes 404 samples of gene expression data from various human cell types exposed to nanoparticles. The datasets in NanoMiner originated from Gene Expression Omnibus (GEO, http://www.ncbi.nlm.nih.gov/geo/) [7], ArrayExpress (http://www.ebi.ac.uk/arrayexpress/) [8], and from our own experiment series [9]. The PRISMA chart [10] in Figure S1 illustrates the acquirement of the data. The nanoparticles studied cover a range of different particle types including metal, metal oxide and carbon-based nanoparticles (Table 1). In addition to ENs, data from studies of particular matter (PM) of various sizes are also included. More specific annotation of each sample can be found in the Table S1 and in the online database. NanoMiner is a versatile toolkit with which the user can analyze and visualize microarray data. The user can browse the sample sets with detailed annotations and sample-wise hierarchical clustering analyses. Further, the user can search for differentially expressed genes with both gene-wise and comparison-wise analysis options. With NanoMiner, it is possible to perform enrichment analysis for a specific gene set to find enriched Gene Ontologies [11] and KEGG [12] pathways. In addition, the user can summarize the gene expression values with several different visualization options. All the data values, analysis results, and sample annotations can be extracted from NanoMiner for further use if necessary. The analysis and visualization options provided within the database are summarized in Figure 1.

**Table 1:** The cell types and the particulate matters used in the datasets in NanoMiner.

Reference	Platform	Cell types	Particulate matter	Samples
(Busch *et al.* 2010) [4] GSE16727	Agilent-014850 Whole Human Genome Microarray 4×44K	human keratinocytes cell line (HaCaT)	WC nanoparticles; WC-Co nanoparticles	40
(Fujita *et al.* 2009) [5] GSE16425	Agilent-014850 Whole Human Genome Microarray 4×44K	human keratinocytes cell line (HaCaT)	ultrafine TiO_2_(T-7); fine TiO_2_(T-20); submicron TiO_2_(T-200)	36
(Gras *et al.* 2009) [6] GSE12405	Agilent-014850 Whole Human Genome Microarray 4×44K	human primary macrophages	carboxilane dendrimer 2G-NN16	6
(Hofer *et al*. 2008) [14] GSE8608	Affymetrix GeneChip Human Genome U133 Plus 2.0	monocyte-derived macrophages (MDM) from chronic obstructive pulmonary disease (COPD) patients and healthy subjects	fine TiO_2_ and ultrafine Printex90	6
(Huang *et al.* 2011) [15] GSE7010	Affymetrix GeneChip Human Genome U133A	human primary epithelial cells	coarse PM (Chapel Hill); fine PM (Chapel Hill); ultrafine PM (Chapel Hill)	12
(Karoly *et al.* 2007) [16] GSE4567	Affymetrix Human GenomeU133 Plus 2.0	human primary pulmonary artery endothelial cells (HPAEC)	ultrafine particle (Chapel Hill)	8
(Kawata *et al.* 2009) [17] GSE14452	Affymetrix Human HG-Focus Target Array	human hepatoma cell line (HepG2)	silver nanoparticles; polysthylene nanoparticles	15
no reference in GEO(E-TABM-679)	Illumina HumanHT-12 v3.0 Expression BeadChip	human lung epithelial cell line (A549)	carbon black; multiwall carbon nanotubes; silica nano; silica micro; silica quartz	36
(Kim *et al.* 2012) [18] GSE20677	Affymetrix GeneChip Human Genome U133 Plus 2.0	human embryonic kidney cell line (293T); human peripheral blood mononuclear cells (PBMC)	Au-nanoparticle EGFP oligonucleotide complex	16
(Tuomela *et al.* 2013)[9] GSE39330	Affymetrix GeneChip Human Genome U219	human monocyte-derived macrophages (HMDM); human monocyte-derived dendritic cells (MDDC); human T cell leukemia-derived cell line (Jurkat)	ZnO-1 (IBU-tec advanced materials AG); ZnO-2 (mandelic acid coated ZnO-1); ZnO-3 (mercaptopropyl-trimethoxysilane coated ZnO-1); ZnO-4 (methoxyl coated ZnO); ZnO-5 (diethylene glycol modified ZnO); ZnO-9 (folic acid modified ZnO)	71
(Tuomela *et al.* 2013)[9] GSE39316	Illumina Sentrix HumanHT-12 Expression BeadChip version 3	human monocyte-derived macrophages (HMDM); human monocyte-derived dendritic cells (MDDC); human T cell leukemia-derived cell line (Jurkat)	ZnO-1 (IBU-tec advanced materials AG); TiO_2_ (Evonik Degussa, Aeroxide® TiO2 p25)	90
(Moos *et al*. 2011) [19] GSE14910	Agilent-014850 Whole Human Genome Microarray 4×44K	human colon cancer cells: CaCo-2, RKO	nanoZnO; nanoFe2O3; nanoSiO2; nanoTiO2; Al2O3; nano-carbon black; microZnO	32
(Moos *et al.* 2011) [19]GSE25167	Agilent-014850 Whole Human Genome Microarray 4×44K	human skin-derived cancer cells(HaCaT and SK Mel-28)	ZnO; TiO_2;_ ZnCl_2;_ ZnO_Transwell	15
(Balakumaran *et al.* 2010) [20] GSE20431	Spotted oligonucleotide; (NIH/CC/DTM) Operon Human Genome Array-Ready Oligo Set (AROS) 4	human bone marrow stromal cells	Gold nanoparticles; FePro	15
(Hanagata *et al.* 2011) [21] GSE33278	Agilent-014850 Whole Human Genome Microarray 4×44K	Lung epithelial cells A549 exposured vs. non-treated cells. Hybridization: 2 replicates. Scanning: 3 replicates	CuO nanoparticles	6

### Experiment Data Visualization and Annotation

**Figure 2:**
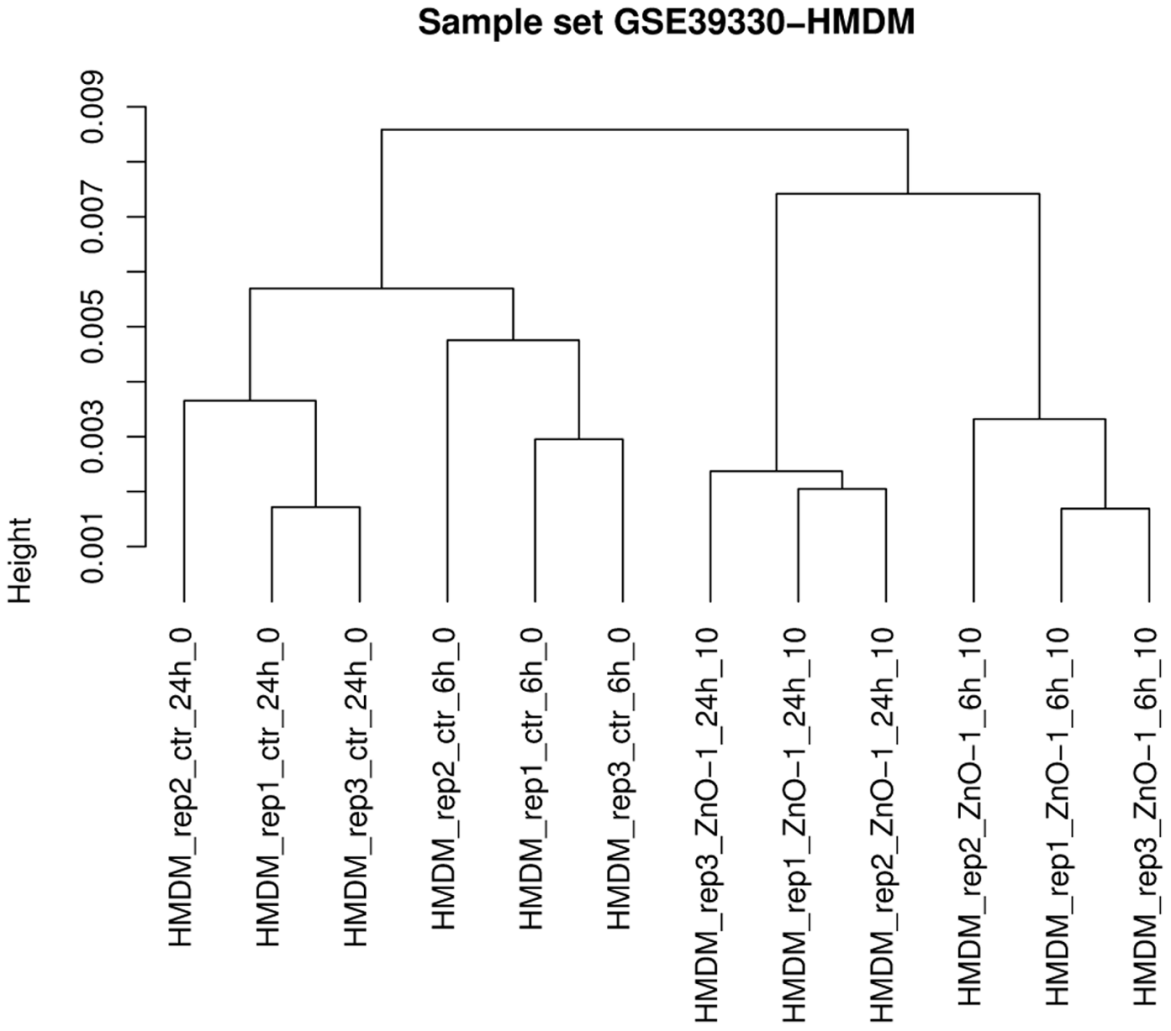
Example of clustering analysis in NanoMiner. The figure shows the clustering of the human monocyte-derived macrophages (HMDM) exposed to 10 µg/ml of ZnO nanoparticles for 6 or 24 hours or left as controls in dataset GSE39330. The clustering was performed using all data (12 samples and 17,617 genes) with Pearson correlation distance and average linkage.

Due to the careful annotation and summarization of each dataset in NanoMiner, it is straightforward to understand the experimental design and the impact of the used treatments. Sample-wise hierarchical clustering has been performed for each set (Figure 2), which shows the natural grouping of the samples, *i.e.* which samples are the most similar or dissimilar. In general, the genome-wide effect of a nanoparticle treatment can be seen in the results of the hierarchical clustering. In the cases where the control samples and the treated samples are grouped into distinct clusters, the treatment has had an effect on large amount of the genes. On the other hand, some treatments may change the expression of only a few genes, in which case the division between the control and treated samples in the clustering result is not that clear. For example, in the dataset GSE39330 [9], the untreated control samples and the ZnO-nanoparticle exposed samples cluster into their own branches, with the sampling timepoint further sub-classifying the samples, instantly revealing the two main parameters affecting the gene expression in this particular sample set (Figure 2).

All the samples in NanoMiner have been annotated in detail. In addition to the information about the samples such as cell type, the annotations, which were manually extracted from the literature, include the experimental parameters such as the type of nanoparticle, dose and exposure time (Table 1, Table S1). These annotations are important for the interpretation of the data and in the generation of new hypotheses to be experimentally tested. Direct links to the original data source are also provided in the annotation section to allow the original data *e.g.* physico-chemical characterization of the nanoparticles to be inspected. Commercial, laboratory synthesized, and ambient particles have been used in the studies included in NanoMiner. In most cases, the nanoparticles have been carefully characterized, which is a prerequisite for detailed interpretation of the toxicity results. However, there is still a need for standardization of reporting of the physico-chemical characterization data [13].

### Identification of Differentially Expressed Genes

To assist in quick identification of a potential gene list that has a dramatic change in expression level when the cells are treated with a certain nanoparticle, we pre-computed the differentially expressed genes within the datasets. Table S2 summarizes the 85 comparisons and the total number of genes measured in each comparison. The differentially expressed gene analysis can be performed either by comparison-wise or gene-wise analysis. In the comparison-wise analysis the user first selects the dataset and the comparison of interest. NanoMiner returns the results of the differential expression analysis including the logarithmic fold change, p-value and adjusted p-value between the comparison groups. By default, a gene is considered to be differentially expressed when it has an adjusted p-value ≤0.05 and an absolute fold change ≥1.5. Further, the results can be downloaded as a.csv file enabling the user to freely select other cut-offs to the gene list. This comparison-wise approach is the traditional approach used in many of the original studies, but since in NanoMiner all the datasets have been consistently preprocessed and systematically annotated, the comparison between the results from multiple datasets is more straightforward than before. This is a unique property of NanoMiner and is not freely available in other web tools at the moment.

In the gene-wise analysis approach, the analysis starts by selecting a list of genes. This list can be directly given by the user, or the genes can be selected based on a specific GO term or a KEGG pathway. Further, the user selects the comparisons of interest, and NanoMiner returns a table with the differential expression analysis results for the selected genes and comparisons (Table 2). With this option, it is easy to detect the nanoparticle treatments that have statistically significantly altered the expression values of the specific genes, GO terms or KEGG pathways, as well as to identify the genes that are differentially expressed in several comparisons. As with the comparison approach, the result table gives the adjusted p-value and the logarithmic fold change value for the genes within each comparison and the analysis results can be downloaded and further analyzed by the user.

**Table 2:** The logarithmic fold changes of the genes when searching for the expression of the most regulated (adj. p<0.001) genes in HMDM sample set of GSE39330 in timepoints 6 h and 24 h within the “GO:0006955: immune response” through other comparisons in NanoMiner.

Ensembl Name	HGNC Name	Ag2CO3_vs_control	Ag_NPs_vs_control	nanoAg_cysteine_vs_control	PS_NPs_vs_control	3d_CoCl_vs_control	3d_WC_Co_vs_control	3h_CoCl_vs_control	PBMCs_AuNP_24 hr_vs_PBMCs_Control	PBMCs_AuNP_48 hr_vs_PBMCs_Control	HMDM_allZnO24	HMDM_allZnO6
ENSG00000056972	TRAF3IP2	NA	NA	NA	NA	0.52	0.06	0.34	−0.55	−1.39*	2.05*	1.88*
ENSG00000090339	ICAM1	1.58*	1.09*	0.92*	0.33	−0.35	−0.49	0.34	−0.26	−1.07*	1.46*	1.35*
ENSG00000095585	BLNK	−0.15	−0.11	−0.04	0	0.54	0.56	0.07	0.34	0.28	−2.00*	−3.61*
ENSG00000103569	AQP9	0.12	0	−0.08	−0.02	−0.09	−0.04	0.14	−2.50*	−4.13*	3.15*	2.34*
ENSG00000112299	VNN1	0.56	0.70*	0.98*	0.59*	0.43	−0.08	0.14	−0.06	−0.08	2.11*	2.31*
ENSG00000112715	VEGF	0.79*	1.16*	1.42*	0.61*	−0.71*	−0.58	−0.08	−1.98*	−2.55*	1.99*	1.50*
ENSG00000124731	TREM1	0.35	0.34	0.16	0.19	−0.01	0.02	0.05	−1.63*	−2.14	4.80*	3.12*
ENSG00000124875	CXCL6	0.1	−0.1	0.13	−0.06	0.06	0.15	0.42	−0.02	−0.08	6.86*	5.39*
ENSG00000134061	CD180	0.02	0.16	−0.12	0.05	0.01	0.11	0	1.07*	1.06*	−2.30*	−2.65*
ENSG00000143641	GALNT2	−0.28	−0.4	−0.39	−0.27	−0.21	−0.64*	1.06*	−0.62	−1.13*	1.50*	1.46*
ENSG00000161574	CCL15	0.16	−0.01	0.06	0.31	−0.11	−0.06	−0.12	−0.9	−0.83	2.14*	3.59*
ENSG00000163734	CXCL3	0.09	0.19	0.2	0.22	0.06	0.13	0.03	−3.10*	−4.35*	2.86*	2.79*
ENSG00000164308	LRAP	−0.3	−0.16	−0.43	−0.21	0.59*	0.07	0.07	−0.18	−0.07	−1.08*	−1.36*
ENSG00000164949	GEM	0.11	0.25	0.18	0.37	0.14	0.01	−0.02	−1.04	−1.56*	2.48*	3.12*
ENSG00000166527	CLEC4D	NA	NA	NA	NA	0.02	−0.06	0.15	−0.83*	−1.17	5.05*	2.34*
ENSG00000187554	FCAR	NA	NA	NA	NA	1.34*	0.39	−0.09	1.62*	1.43*	−3.63*	−2.55*
ENSG00000186431	TLR5	0.13	0.02	0.07	0.05	NA	NA	NA	−0.48	−1.19	2.98*	2.27*

The genes (rows) with the absolute fold change ≥1.5 (or 0.585 on log base 2 scale) and an adjusted p-value ≤0.05 from the differential expression analysis is marked with * in the given comparison (column).

Based on the number of differentially expressed genes and the magnitude of the changes in each comparison, the conditions in which the selected pathway is most strongly affected by the treatment can be detected. In the example dataset [9], human monocyte-derived macrophage (HMDM) samples of GSE39330, there are 2110 and 2366 differentially expressed genes at the 6 and 24 hour timepoint, respectively, with default adjusted p-value ≤0.05 and an absolute fold change ≥1.5 (Table S3). Previously, ZnO nanoparticles were reported to induce unfolded protein response in colon and skin cancer cell lines [14]. To investigate with NanoMiner if this pathway is also affected in HMDM, we searched differentially expressed genes based on the GO term GO:0006986 “response to unfolded protein” in the GSE39330 HMDM comparisons. The analysis revealed that 30% and 36% of the genes within the GO:0006986 were detected as differentially expressed with p-value ≤0.05 and absolute fold change ≥1.5 after ZnO-treatment at the 6 and the 24 hour timepoints, respectively (Table S4).

### Enrichment Analysis

NanoMiner also provides an option to perform an enrichment analysis. With this tool, it is possible to find KEGG pathways and GO terms that are enriched within the group of genes. This gene group can be provided by the user, or it can, for example, be the result of a differential expression analysis in the database. NanoMiner computes the enriched KEGG pathways and GO terms and returns the results including the p-value, the ratio of the enrichment, the number of detected genes and the expected number of genes within a given pathway or GO term. Further, NanoMiner provides illustrations of the enriched KEGG pathways where the genes from the input gene list are colored in pink while the other genes within the pathway are marked in green.

When KEGG pathway enrichment analysis is performed on the differentially expressed genes detected in our example HMDM sample set of GSE39330 at the 6 h and the 24 h timepoints, the p53 KEGG pathway is found to be enriched (Figure S2). Similarly, the GO term “GO:0006955: immune response” is found to be enriched (Table S5). We can select the genes belonging to this GO term, and study how these genes are regulated in the other datasets in NanoMiner by searching for them in the comparison lists, available under the “Differential Expression” section in the database. NanoMiner returns a table in which the regulated genes in this GO term are highlighted in each dataset (Table S6). Based on the number of differentially expressed genes, the comparisons in which the genes in the selected GO class have been substantially regulated can be identified. As shown in Table 2, many of the selected “immune response” genes are also regulated in the GSE20677 dataset reported by Kim *et al.* [18] in a study of human peripheral blood mononuclear cells.

### Visualization and Analysis Tools

**Figure 3:**
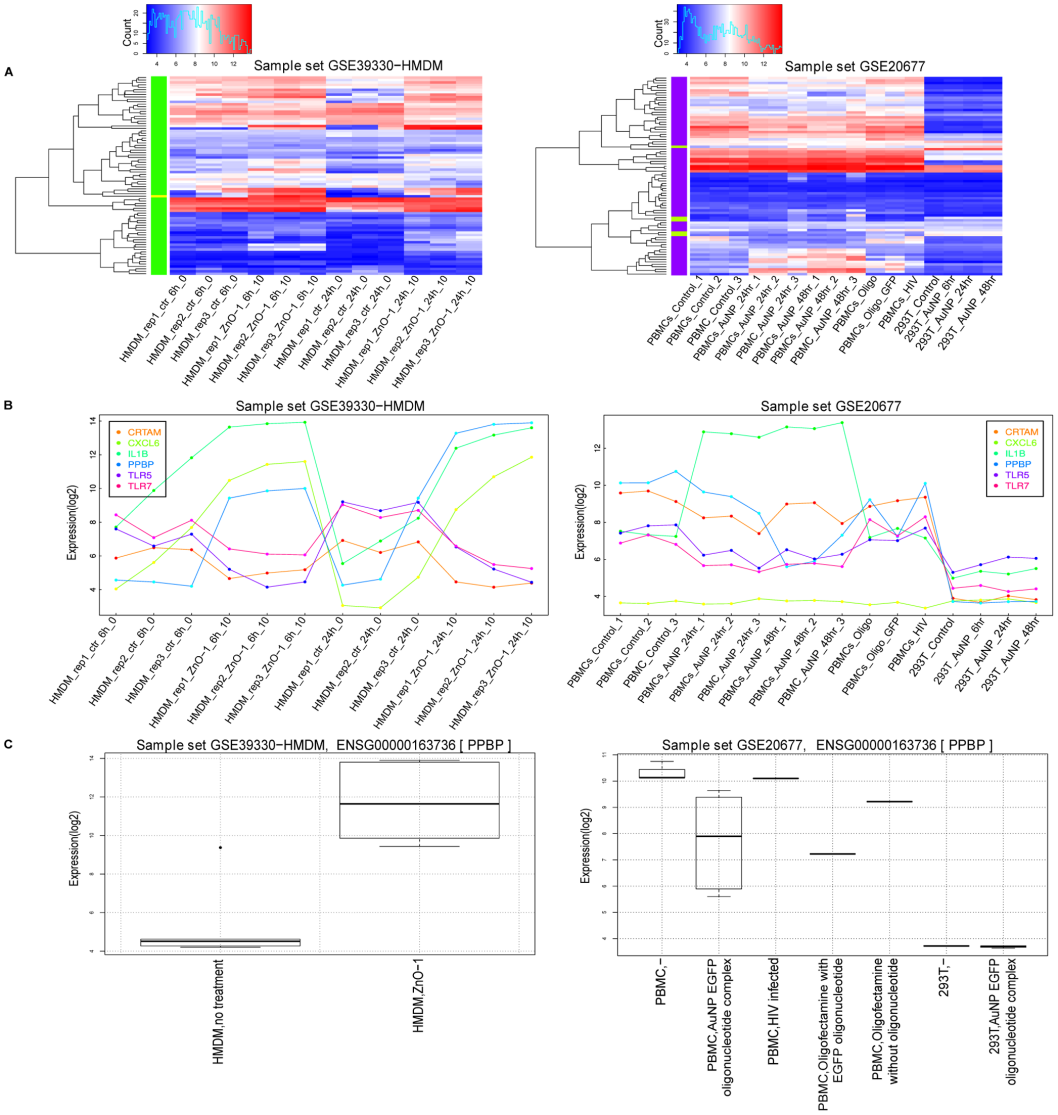
Gene-centric visualization options in NanoMiner. 82 genes (Table S6) from “GO:0006955: immune response” were detected as differentially expressed genes in HMDM cells treated with 10 µg/ml ZnO-nanoparticle compared to untreated control cells at 24 hours in dataset GSE39330. **A)** The heatmap presentation of the expression of these genes in GSE39330 and GSE20677. **B)** The expression profile of the three most highly upregulated (PPBP, CXCL6, IL1B) and downregulated genes (CRTAM, TLR7, TLR5) after exposing HMDM cells with 10 µg/ml of ZnO-nanoparticle for 6 hours belonging to GO term GO:0006955: immune response plotted over all the samples in GSE39330 and GSE20677. **C)** Boxplots of the expression level of gene PPBP in the datasets GSE39330 and GSE20677 by treatment.

Studying how a particular gene or a group of genes behave in a given condition is often an important starting point for an experimental biologist to construct a hypothesis and the experimental setup. To facilitate this, NanoMiner provides several ways to visualize and analyze the gene-centric data. All visualization tools, available under the “Gene Search” section, show how a treatment affects gene behavior at the expression level. Heatmaps (Figure 3A) show the expression pattern of selected genes in a given dataset. The expression plots visualize the log_2_-transformed gene expression values across the samples in the datasets (Figure 3B). In addition, boxplots (Figure 3C) summarize the expression distributions across the samples. The researcher can draw boxplots by grouping the samples based on the treatment, replicate or cell type used in the selected dataset depending on the biological question to be solved. The versatility of the visualization tools in NanoMiner allows the user to search for and compare different relationships in the data, which is not possible with the other tools.

## Materials and Methods

### Dataset Collection

**Figure 4:**
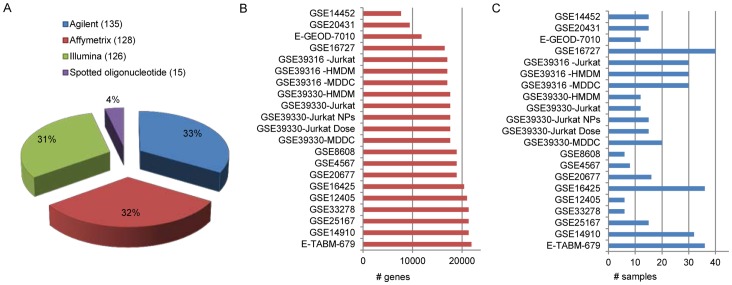
Data in NanoMiner. **A)** The samples in NanoMiner measured with different platforms, **B)** Number of genes measured in the datasets in NanoMiner, **C)** Number of samples in each dataset.

The datasets in NanoMiner were downloaded as raw data files from Gene Expression Omnibus (GEO, http://www.ncbi.nlm.nih.gov/geo/) [7] and ArrayExpress (http://www.ebi.ac.uk/arrayexpress/) [8]. The datasets were included in NanoMiner if they are measured with Affymetrix, Agilent, Illumina or Spotted Oligonucleotide platforms (Figure 4A) and composed of at least 6 human samples treated with nanoparticles (Figure 4B–C). Further, to assure that the datasets are from nanoparticle-related studies that provide sufficient description of the experimental set up, all the publications were also manually inspected. The detailed annotations of the selected datasets [4]–[6], [14]–[21] are listed in the Table 1.

### Data Pre-processing

All the data values in NanoMiner are preprocessed before imported into the system. R [22] and Bioconductor [23] were used as the main tool for pre-processing and analysis of all the datasets in the database. Since the data in NanoMiner came from more than one platform, different R packages and pre-processing methods were used. Datasets measured with Affymetrix platforms were pre-processed using the Robust Multi-array Average (RMA) [24] algorithm in the R package *affy* [25]. In addition, we used the Brainarray CDF-files [26] (Version 14) with which we analyzed all the values directly from probes to ENSEMBL [27] genes, and thus the data did not need to be linked separately for each Affymetrix array generation from probesets to genes. The R package *lumi* [28] and the quantile normalization algorithm were used for pre-processing the datasets from Illumina platform. Further, the Illumina IDs were directly linked to ENSEMBL genes with the *lumiHumaAll.db* package. From the Agilent platform and spotted oligonucleotide arrays, the samples were pre-processed with *limma* package [29]. The samples were first background corrected with the normexp method [30]. Then the two-channel samples were normalized with the loess method and the one-channel samples with quantile normalization. The probe IDs were linked to ENSEMBL genes with *biomaRt* [31] package.

To reveal the relationships between the samples within a dataset, all the gene expression values were clustered using hierarchical clustering with Pearson correlation distance and average linkage. The R package *amap* [32] was used to create the hierarchical clustering figures.

### Expression Profiling

The R package *gplot* and *amap* were used for producing an expression profile, boxplots and heatmap for a given gene or a gene set. The expression profile visualizes the log_2_-transformed values of the selected gene in the datasets. In the heatmaps, the gene expression values (rows) are clustered using correlation distances with average linkage. The columns represent the samples. The red and blue colors denote high and low expression values, respectively. In the boxplots, the top and bottom sides of the box represent the upper and lower quartiles (75th and 25th percentiles) respectively, while the band within the box represents the median (50th percentile). The whiskers represent the most extreme data points, and *n* is the total number of samples within each group. In every profiling option of NanoMiner, all the gene values can be downloaded for further use if needed.

### Differentially Expressed Gene Analysis

Linear modeling and empirical Bayes methods, which were implemented in the R package *limma* [33], [29], were used to detect the differentially expressed genes between two groups, in this case, the control and treatment sample groups. Raw p-values were adjusted using the Benjamini and Hochberg multiple adjustment method [34]. Genes with an adjusted p-value ≤0.05 and an absolute fold change ≥1.5 were considered significantly differentially expressed. The differentially expressed genes were pre-computed based on all the possible comparisons between the treatment and control sample groups, and the results were stored into the NanoMiner database. The differential gene expression analyses were performed within sample sets, and thus the uneven distribution of particles in NanoMiner will not have effects on analysis results across sample sets. From the differential expression analysis, all the fold changes with the p-values and adjusted p-values can be retrieved. In addition, the analysis retrieves the B-values of the comparisons and for one channel arrays also the average expression of the genes within the dataset. All the results of the differential expression analysis are downloadable from the database as.csv sheets.

### Enrichment Analysis

Enrichment analysis is a tool to find enriched GO terms or KEGG pathways for a given gene set. The *GOStats* package [35] and *KEGG.db* which are used in the analysis utilize the hypergeometric distribution to obtain enriched ontologies and pathways. By default, all human ENSEMBL genes are used as a background gene group and the categories with a p-value ≤0.05 are considered significantly enriched.

### Web-based Database Construction

NanoMiner is developed using CakePHP and MySQL on the Linux platform with Apache as the web server (Figure S3). Its web-based interface uses PHP and HyperText Markup Language (HTML) forms for most of the input and output but also uses some Java-based applets. It also uses the R as a supplemental tool for making queries, analyzing data and drawing illustrations in the database as a background process. The pre-processed data are stored using the MySQL relational database management system, which supports the structured query language (SQL) standard.

### Future Development of NanoMiner

As transcriptomics experiments are highly informative in nanotoxicological studies, there will be many new studies available in the future. We thus intend to update NanoMiner regularly to include the data of the latest studies. Users can suggest new data to be added into NanoMiner under the “Feedbacks” section or by directly contacting us at nanominer@cs.tut.fi. In addition, NanoMiner will be developed to follow the new standards and requirements of the field based on the suggestions from the users and the scientific community.

## Discussion

NanoMiner is a unique and urgently needed database of transcriptomics data gathered in the nanosafety research field containing hundreds of samples of nanoparticle-exposed cells analyzed by a variety of microarray platforms. With the several analysis and visualization options, NanoMiner can systematically reveal the effects of various nanoparticle exposures on the levels of gene expression, KEGG pathways and Gene Ontologies. NanoMiner is the first database that can be used to directly address the questions raised by the users, including: which are the genes whose expression values are statistically significantly changed during nanoparticle exposures for various types of cells, and which are the nanoparticles that have an effect on the expression of a specific gene. To obtain answers to these questions without NanoMiner, it would be compulsory to go through every study one by one and to accept the fact that all the studies could have been preprocessed, normalized and analyzed with different methods, which in turn will cause variation in the results and possibly convey to misleading interpretations. With the systematic annotation and analysis in NanoMiner, nanoparticle related transcriptomics research becomes much more feasible and straightforward. NanoMiner has been designed to be intuitive and easy to use, which makes utilization of the data fast and simple. The database is an important step towards harmonization of the information gathered in nanoparticle research.

The NanoMiner web resource (http://nanominer.cs.tut.fi/) is freely available upon registration for academic researchers using the service for noncommercial purposes. We anticipate that there will be new transcriptomics studies available in the future, and thus intend to regularly process and integrate the latest results into NanoMiner, in order to provide a durable resource for the nanomaterial research community.

## Supporting Information

Figure S1 **PRISMA flow chart for demonstrating the selection of the samples to be included in NanoMiner.** We initially identified 361 samples through public databases, and 161 samples were identified from other sources. 118 samples were excluded because the studies are either not related to nanoparticles, or there are too few samples in the study in question.
(TIF)

Figure S2 **Illustration of the enriched KEGG P53 Signaling pathway.** KEGG P53 Signaling pathway was enriched within the differentially expressed genes (DEGs) detected in HMDM sample set of GSE39330 in both timepoints 6 h and 24 h. The DEGs are highlighted in pink.
(TIF)

Figure S3 **The system architecture of NanoMiner.** The Web Browser sends requests to the Apache Web Server from the end users. Apache Web Server receives and processes the requests from the Web browser, loading the PHP scripts for the PHP scripting engine. The scripting engine parses and executes the scripts. PHP engine communicates with the MySQL database and fetches the data from the database. The database with MySQL management system executes the SQL statements and returns the query results, and then the web server materializes the results further and sends back to the end users.
(TIF)

Table S1 **Annotation table of the samples in NanoMiner.** The annotations include GEO ID/Array Express ID, Platform information, description of preprocessing and normalization methods, summary of the dataset, citation, pubmed ID, and the detailed information of the treatment i.e. timepoint, nanoparticle dose, and nanoparticle size.
(XLS)

Table S2 **List of comparisons for which the differentially expressed gene lists are available.** The table shows the information about the 85 comparisons that can be uploaded from NanoMiner, including the dataset ID, name of the comparison, number of genes in the array, information of the treated and control samples, and the cell type of the samples.
(XLS)

Table S3 **Results of the differential expression analysis for the ZnO 10 µg reatments for the 6** **h and the 24** **h timepoints of HMDM samples within the data set GSE39330.** Table shows the results returned from NanoMiner. Results include the logarithmic fold change of the gene values between the treatment and the control samples, average value across all the samples, p-value and adjusted p-value for the gene showing whether the difference in the gene value between the gene groups is statistically significant, and B-statistics that is a log odd value showing the probability that the gene is differentially expressed.
(XLS)

Table S4 **The adjusted p-values of the genes within the GO:0006986 “response to unfolded protein” showing whether the gene is differentially expressed in the two comparisons of the ZnO treatments of HMDM samples within the data set GSE39330.** By default, when the adjusted p-value ≤0.05 the gene is considered to be differentially expressed between the treated and control samples.
(XLS)

Table S5 **The enrichment analysis results for the differentially expressed genes of GSE39330 HMDM samples.** The genes differentially expressed (adjusted p-value ≤0.05 and absolute fold change ≥1.5) at both timepoints were input to the enrichment analysis, and the enrichments were computed through the KEGG pathways, GO Biological Processes, GO Molecular Functions, GO Cellular Components. The result gives the p-value showing whether the term is significantly enriched, expected count of the genes within the term, the odds-ratio of the genes of the list within the term, the total number of the genes and the genes found in the list within the term.
(XLS)

Table S6 **The adjusted p-values of the gene-wise differential expression analysis for the genes within the GO:0006955: immune response.** The value is marked in red, if the adjusted p-value ≤0.05 and the absolute fold change between the treated and control sample value ≥1.5.
(XLS)
