# Supplementary figures and images for: NanoMiner — Integrative Human Transcriptomics Data Resource for Nanoparticle Research
Source: PLoS One. 2013 Jul 12;8(7):e68414. doi: 10.1371/journal.pone.0068414 (PMC3709991; doi:10.1371/journal.pone.0068414)

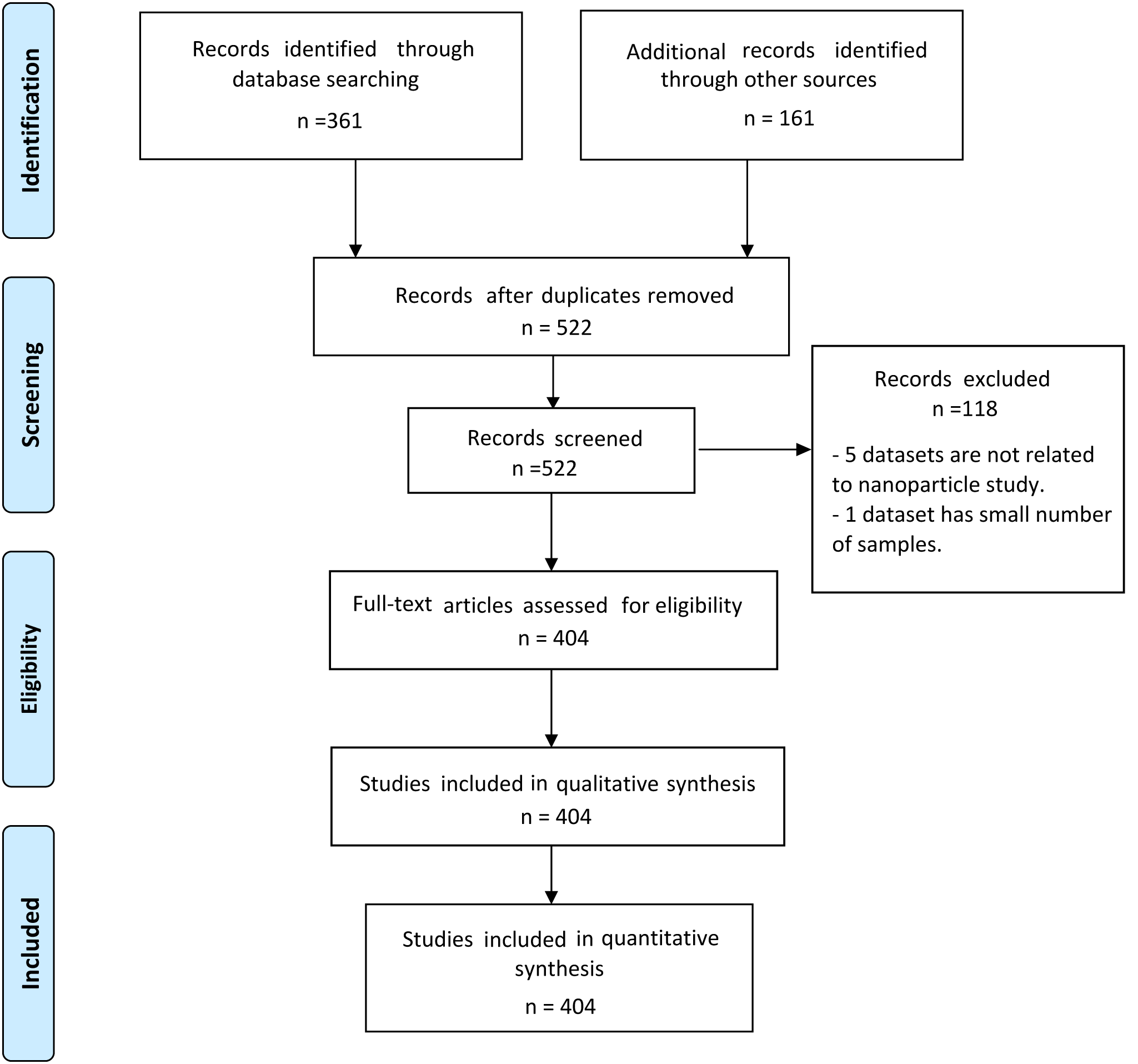

Supplement: Figure S1 — PRISMA flow chart for demonstrating the selection of the samples to be included in NanoMiner. We initially identified 361 samples through public databases, and 161 samples were identified from other sources. 118 samples were excluded because the studies are either not related to nanoparticles, or there are too few samples in the study in question. (TIF) [file pone.0068414.s001.tif]

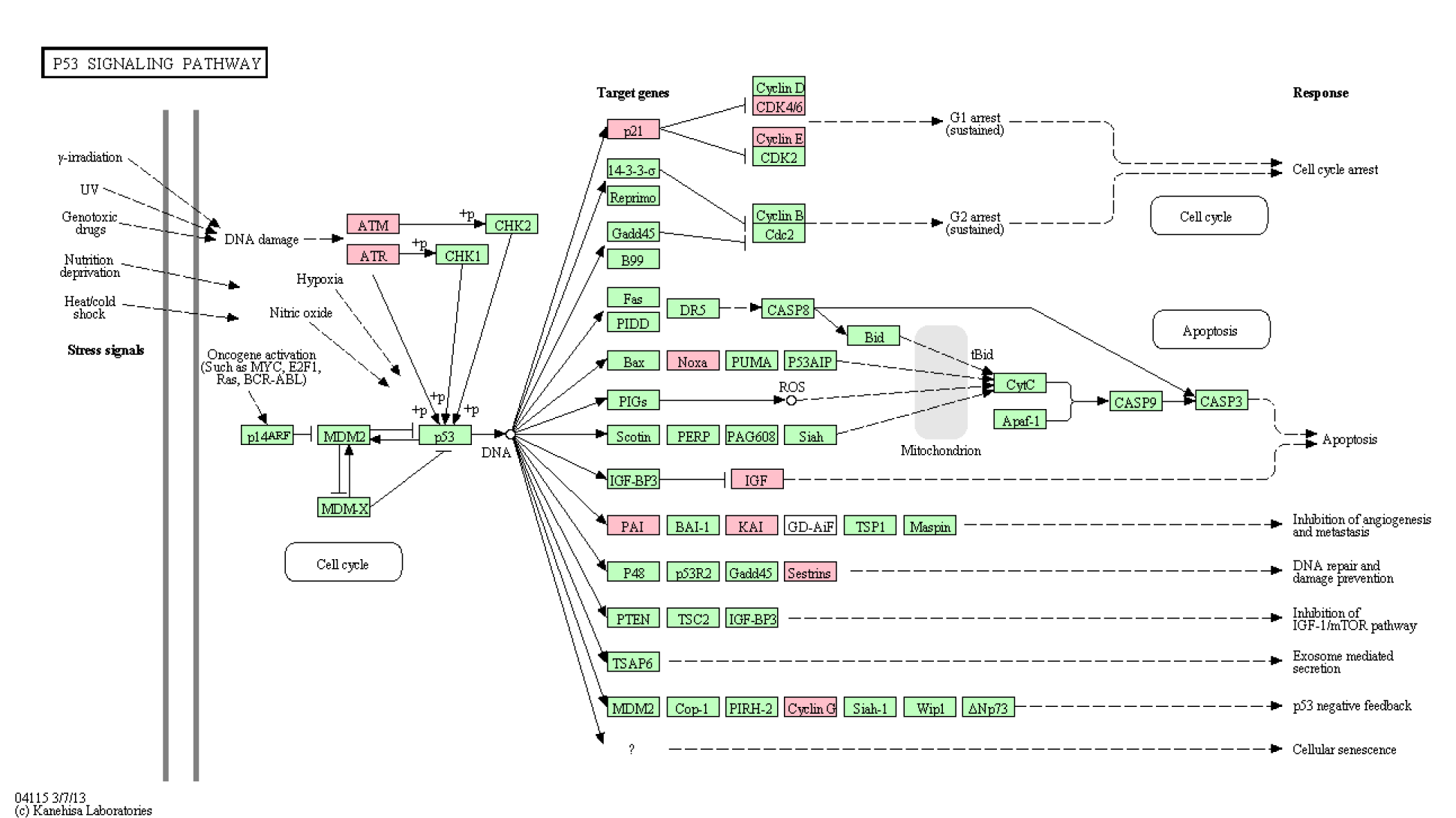

Supplement: Figure S2 — Illustration of the enriched KEGG P53 Signaling pathway. KEGG P53 Signaling pathway was enriched within the differentially expressed genes (DEGs) detected in HMDM sample set of GSE39330 in both timepoints 6 h and 24 h. The DEGs are highlighted in pink. (TIF) [file pone.0068414.s002.tif]

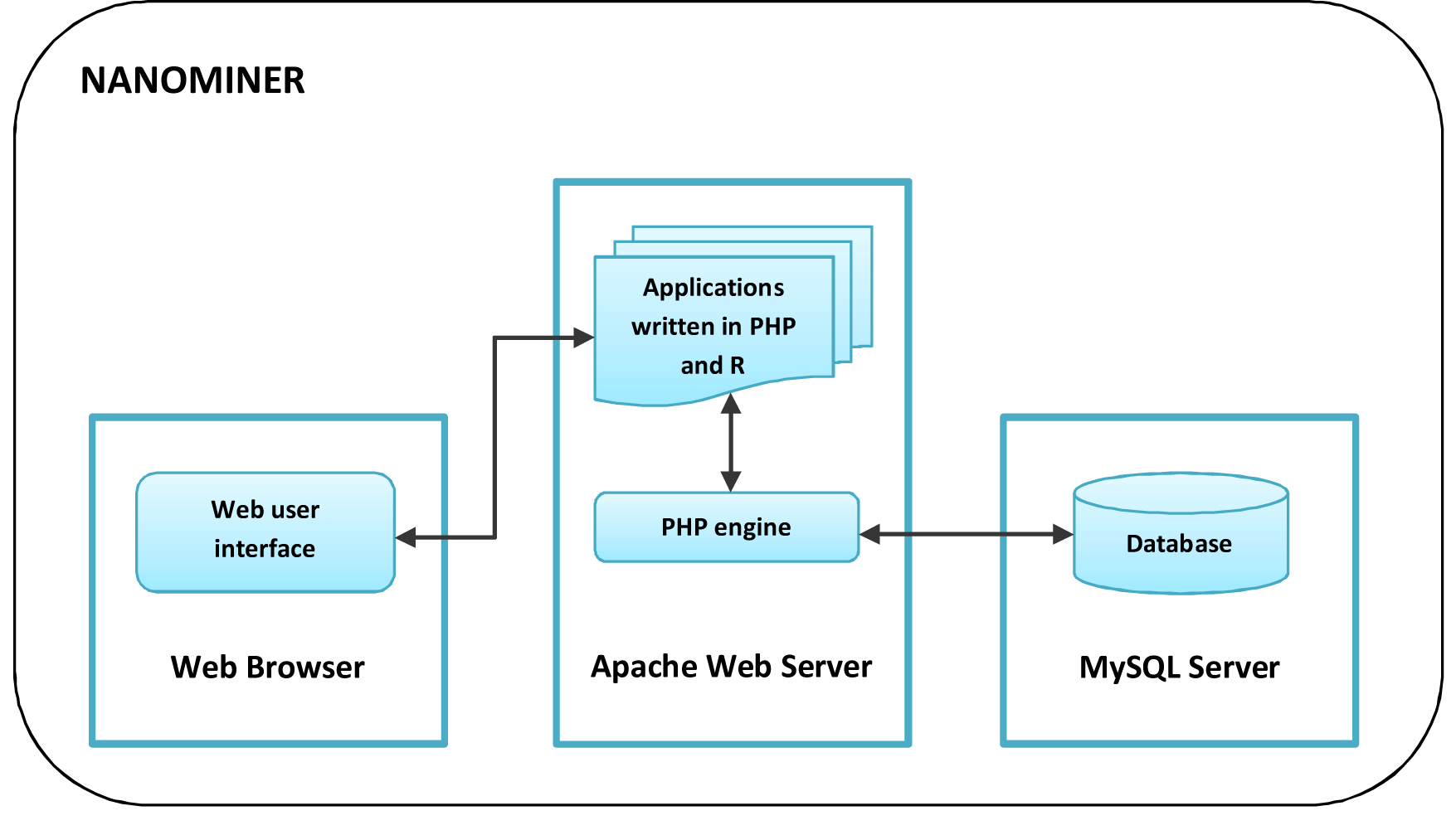

Supplement: Figure S3 — The system architecture of NanoMiner. The Web Browser sends requests to the Apache Web Server from the end users. Apache Web Server receives and processes the requests from the Web browser, loading the PHP scripts for the PHP scripting engine. The scripting engine parses and executes the scripts. PHP engine communicates with the MySQL database and fetches the data from the database. The database with MySQL management system executes the SQL statements and returns the query results, and then the web server materializes the results further and sends back to the end users. (TIF) [file pone.0068414.s003.tif]
